# Music for Invalids

**Published:** 1871-02

**Authors:** 


MUSIC FOR INVALIDS.—Music as an agent for promoting health is of high value. If invalids would devote an hour or two daily to practicing vocal music, it would often restore them to health. Persons with weak lungs may thus ward off fatal lung disease. The effect on both body and mind are excellent.—*Herald of Health.*

